# Identification of a *RAC/AKT*-like gene in *Leishmania* parasites as a putative therapeutic target in leishmaniasis

**DOI:** 10.1186/s13071-017-2379-y

**Published:** 2017-10-10

**Authors:** Rubén E. Varela-M, Rodrigo Ochoa, Carlos E. Muskus, Antonio Muro, Faustino Mollinedo

**Affiliations:** 1 Instituto de Biología Molecular y Celular del Cáncer, Centro de Investigación del Cáncer, Consejo Superior de Investigaciones Científicas (CSIC)-Universidad de Salamanca, Campus Miguel de Unamuno, Salamanca, Spain; 2 Facultad de Ciencias Básicas, Universidad Santiago de Cali, Campus Pampalinda, Santiago de Cali, Colombia; 3 Programa de Estudio y Control de Enfermedades Tropicales (PECET), Universidad de Antioquia, Medellín, Colombia; 4 Laboratorio de Inmunología Parasitaria y Molecular, IBSAL-CIETUS, Facultad de Farmacia, Universidad de Salamanca, Campus Miguel de Unamuno, Salamanca, Spain; 5 Laboratory of Cell Death and Cancer Therapy, Department of Cellular and Molecular Medicine, Centro de Investigaciones Biológicas, CSIC, C/ Ramiro de Maeztu 9, E-28040 Madrid, Spain

**Keywords:** PKB/AKT, RAC/AKT-like, Therapeutic target, *Leishmania*, *Leishmania donovani*, Trypanosomatid

## Abstract

**Background:**

Leishmaniasis is one of the world’s most neglected diseases caused by at least 20 different species of the protozoan parasite *Leishmania*. Although new drugs have become recently available, current therapy for leishmaniasis is still unsatisfactory. A subgroup of serine/threonine protein kinases named as related to A and C protein kinases (RAC), or protein kinase B (PKB)/AKT, has been identified in several organisms including *Trypanosoma cruzi* parasites. PKB/AKT plays a critical role in mammalian cell signaling promoting cell survival and is a major drug target in cancer therapy. However, the role of protozoan parasitic PKB/AKT remains to be elucidated.

**Results:**

We have found that anti-human AKT antibodies recognized a protein of about 57 kDa in *Leishmania* spp. parasites. Anti-human phospho-AKT(Thr308) antibodies identified a protein in extracts from *Leishmania* spp. that was upregulated following parasite exposure to stressful conditions, such as nutrient deprivation or heat shock. Incubation of AKT inhibitor X with *Leishmania* spp. promastigotes under stressful conditions or with *Leishmania*-infected macrophages led to parasite cell death. We have identified and cloned a novel gene from *Leishmania donovani* named *Ld-RAC/AKT-*like gene, encoding a 510-amino acid protein of approximately 57.6 kDa that shows a 26.5% identity with mammalian AKT1. *Ld*-RAC/AKT-like protein contains major mammalian PKB/AKT hallmarks, including the typical pleckstrin, protein kinase and AGC kinase domains. Unlike mammalian AKT that contains key phosphorylation sites at Thr308 and Ser473 in the activation loop and hydrophobic motif, respectively, *Ld*-RAC/AKT-like protein has a Thr residue in both motifs. By domain sequence comparison, we classified AKT proteins from different origins in four major subcategories that included different parasites.

**Conclusions:**

Our data suggest that *Ld*-RAC/AKT-like protein represents a *Leishmania* orthologue of mammalian AKT involved in parasite stress response and survival, and therefore could become a novel therapeutic and druggable target in leishmaniasis therapy. In addition, following comparative sequence analyses, we found the RAC/AKT-like proteins from *Leishmania* constitute a subgroup by themselves within a general AKT-like protein family.

**Electronic supplementary material:**

The online version of this article (10.1186/s13071-017-2379-y) contains supplementary material, which is available to authorized users.

## Background

Leishmaniasis, caused by at least 20 species of the protozoan parasite *Leishmania*, is classified in three different clinical forms, visceral, cutaneous and mucocutaneous leishmaniasis, which have different immunopathologies and degrees of morbidity and mortality. Visceral leishmaniasis, which results in splenomegaly and hepatomegaly, is caused by *L. donovani* and *L. infantum* and is fatal if not treated. Leishmaniasis is a major health problem in many parts of the world, affecting 12 million people worldwide, mainly in developing countries; 350 million people are considered at-risk of contracting the disease, and some two million new cases occur yearly [1]. In addition, increasing immigration, tourism, and military activity in *Leishmania* endemic areas have posed a risk, threatening to expand the disease to nonendemic areas of the world. Major challenges for antileishmanial chemotherapy include the availability of few drugs, emergence of drug resistance, toxicity and lack of cost-effectiveness analysis. The availability of the complete genome sequence of various species of *Leishmania*, including *L. major*, *L. infantum*, *L. braziliensis*, *L. donovani*, *L. mexicana*, *L. amazonensis* and *L. panamensis* [2–7] represents an extraordinary resource for the discovery of new antileishmanial targets. The comparison of the parasite genome with the human genome sequence facilitates the identification of *Leishmania-*specific genes for which drugs could be developed, thereby limiting the chance for interaction with human homologues. However, most of the genes found in *Leishmania* parasites remain to be characterized, and less than half of annotated genes have been assigned gene ontology terms.

The serine/threonine kinase protein kinase B (PKB) or AKT, a member of the AGC family of serine/threonine kinases, is an important regulator of cell proliferation and survival in mammalian cells. Data accumulated in the last decade have established that AKT also plays a major role in cancer development and progression, prompting the development of drugs targeting this survival pathway in cancer therapy [8–11]. The structure of AKT comprises three conserved domains: an N-terminal pleckstrin homology (PH) domain, which binds phosphoinositides with high affinity; a central catalytic domain; and a C-terminal regulatory domain [12]. AKT has a wide range of cellular substrates, and the oncogenicity of AKT arises from activation of both proliferative and anti-apoptotic signaling, thus making this kinase an attractive target for cancer therapy. Activation of mammalian AKT depends on its recruitment to the membrane through binding of phosphatidylinositol-3,4,5-trisphosphate (PIP3) to the PH domain of AKT, and subsequent phosphorylation at two key residues, Thr308 and Ser473, located at the catalytic domain and C-terminal regulatory domain, respectively [13]. The physiological action of AKT kinase is mediated through the phosphorylation of a wide variety of downstream substrates [12–15]. One of the major AKT substrates is glycogen synthase kinase-3 (GSK-3), which has been identified in *Leishmania major* and *Trypanosoma brucei* and has been suggested as a potential drug target in trypanosomatids [16–18]. The fast adaptability of *Leishmania* parasites to different adverse environments in their life-cycle, including changes in temperature, pH, and nutrient availability, suggests these protozoa possess effective mechanisms to ensure survival in the face of these challenges. Because glycogen synthase kinase-3 has been identified in *Leishmania* parasites and is a substrate of the cell survival molecule AKT [19], this prompted us to investigate the putative presence of an AKT homologue in *Leishmania* parasites that might represent a potential drug target. A subgroup of Ser/Thr protein kinases, related to protein kinases A and C (RAC) and to PKB/AKT, has been identified in a number of mammalian cells [20], *Drosophila melanogaster* [21], *Caenorhabditis elegans* [22], *Dyctiostelium discoideum* [23], *Entamoeba histolytica* [24], *Giardia intestinalis* [25], and *Trypanosoma cruzi* [26]. However, no AKT proteins have been so far reported in *Leishmania* parasites.

Here, we show that *Leishmania* spp. express AKT-like genes. We cloned and sequenced a novel gene, named *Ld-RAC/AKT*-like gene, from *Leishmania donovani* (MHOM/IN/80Dd8) that encoded a protein closely related to putative or previously reported RAC serine-threonine kinases from other *Leishmania* and *Trypanosoma* species, as well as to mammalian AKT. Our data show that *Ld*-RAC/AKT-like protein may behave as a survival molecule in *Leishmania* parasites, and could become a novel target for leishmaniasis therapy.

## Methods

### Cell culture

The *Leishmania* strains used in this study were: *L. panamensis* (MHOM/CO/87/UA140), *L. infantum* (MCAN/ES/96/BCN150), *L. donovani* (MHOM/IN/80/Dd8), and *L. braziliensis* (MHOM/CO/88/UA301). *Leishmania* promastigotes were grown at 26 °C in the RPMI-1640 culture medium, containing 10% heat-inactivated fetal bovine serum (FBS), 2 mM L-glutamine, 100 U/ml penicillin, and 100 μg/ml streptomycin. Promastigotes in the stationary growth phase were used for macrophage infection. These were prepared by incubating a starting inoculum of 1 × 10^6^ parasites/ml for 5–6 days. For the experiments of nutritional stress, the culture medium was prepared as above in the absence of FBS. For the thermal shock assays, parasites were incubated for 3 h at 37 °C in the complete culture medium as above.

### Western blot

1.5 × 10^7^ promastigotes were lysed with 180 μl of 150 mM NaCl, 10 mM HEPES, 1% CHAPS, and 0.1 mM sodium orthovanadate, in the presence of protease inhibitors (1 mM phenylmethylsulfonyl fluoride, 20 μg/ml aprotinin, and 20 μg/ml leupeptin). Proteins (40–60 μg) were subjected to SDS polyacrylamide gel electrophoresis under reducing conditions and transferred to PVDF membranes (Merck Millipore, Billerica, MA, USA). Membranes were blocked with 5% (*w*/*v*) skim milk powder in 50 mM Tris-HCl (pH 8.0), 150 mM NaCl and 0.1% (*v*/*v*) Tween 20 (TBST) for 90 min at room temperature, and then incubated for 1 h at room temperature or overnight at 4 °C with the following primary antibodies: anti-60 kDa phospho-AKT (pAKT) (Thr308) and anti-60 kDa pAKT (Ser473) rabbit polyclonal antibodies, which recognize the phosphorylated forms at Thr308 and Ser473 respectively in human AKT (Cell Signaling Technology, Beverly, MA, USA) (1:1000 dilution in TBST with 5% BSA), and the anti-60 kDa AKT rabbit polyclonal antibody (H-136) (1:1000 dilution in TBST with 5% BSA) that recognizes the whole human protein AKT1/2/3 (Santa Cruz Biotechnology, Santa Cruz, CA, USA). Antibody reactivity was monitored with horseradish peroxidase (HRP)-conjugated anti-rabbit IgG, using an enhanced chemiluminescence detection kit ECL-PLUS (GE Healthcare Life Sciences, Piscataway, NJ, USA). Both Fujifilm super RX autoradiography films (Tokyo, Japan) and image capture by Odyssey imaging system (LI-COR Biosciences, Lincoln, NE, USA) were used to visualize immunoreactive bands. The molecular weight of the immunoreactive bands was determined using molecular weight standard markers (Precision Plus Protein™ standards, BioRad, Hercules, CA, USA).

### Flow cytometry determination of apoptosis-like cell death

*Leishmania* spp. promastigotes (2 × 10^6^) were treated as indicated, and then parasites were centrifuged (1000 × *g*, 5 min), and analyzed for apoptosis-like cell death as previously described after propidium iodide staining [27]. The induction of apoptosis was monitored as the appearance of the sub-G_0_/G_1_ phase (hypodiploid cells) in cell cycle analysis, indicating DNA breakdown [27, 28]. Apoptotic cells were quantified as the percentage of cells in the sub-G_0_/G_1_ region (hypodiploid DNA content) following cell cycle analysis [27, 28], using a fluorescence-activated cell sorting (FACS) Calibur flow cytometer (Becton Dickinson, San Jose, CA, USA). Data were analyzed with WinMDI 2.8 software (Scripps Institute, San Diego, CA, USA).

### Infection of J774 macrophages with *L. panamensis*

J774 macrophage-like cells were cultured at the exponential growth phase (3 × 10^5^ cells/ml) in complete RPMI-1640 medium (containing 10% FBS, 2 mM L-glutamine, 100 U/ml penicillin, 100 μg/ml streptomycin), at 37 °C in a humidified air/CO_2_ incubator (95:5, *v*/*v*), and then infected overnight with stationary-phase *L. panamensis* promastigotes (1/10 macrophage/promastigote ratio). Non-internalized promastigotes were washed out (3 PBS washes). *Leishmania*-infected macrophages were treated with 10 μM AKT inhibitor X (10-(4′-(N-diethylamino)butyl)-2-chlorophenoxazine) (Calbiochem, San Diego, CA, USA) for 24 h and then stained with Giemsa to calculate the number of intracellular amastigotes in 100 infected macrophages. In addition, untreated control and AKT inhibitor X-treated infected macrophages were pelleted, and the percent of apoptotic macrophages was analyzed by flow cytometry as indicated above.

### mRNA isolation and cDNA synthesis in *L. donovani*

Total RNA from 1 × 10^7^ *L. donovani* (MHOM/IN/80/Dd8) promastigotes was isolated using the RNeasy mini kit (Qiagen, Hilden, Germany), following the manufacturer’s instructions. RNA preparations were carefully checked by gel electrophoresis and found to be free of DNA contamination. Total RNA (2 μg), primed with 1 μM oligo-(pdT)_15_, was reverse-transcribed into cDNA with 10 units AMV reverse transcriptase (Hoffmann-La Roche, Basel, Switzerland), according to the manufacturer’s instructions for 1 h at 42 °C in a final volume of 20 μl. To evaluate the quality of the generated cDNA, we amplified the constitutively expressed gene *kmp-11* (GenBank/EMBL accession no. XM_003864757) as previously described [29]. KMP11 protein is one of the most abundant molecules on the cell surface of *Leishmania* spp*.* parasites*.* The sense and antisense primers for *kmp11* gene cDNA amplification were 5′-ATG GCC ACC ACG TAC GAG GAG-3′ and 5′-GGA CGG GTA CTG CGC AGC CTT-3′. PCR was performed in a GeneAmp PCR system model 9600 (Perkin-Elmer, Norwalk, CT, USA). PCR amplification was as follows: 1 cycle at 95 °C for 5 min as an initial denaturation step, then denaturation at 95 °C for 1 min, annealing for 1 min at 58 °C, and extension at 72 °C for 1 min (30 cycles). PCR products (236 bp) were electrophoresed on 2% agarose gels in 1× TAE buffer (40 mM Tris-acetate, 1 mM EDTA (pH 8.0)) and visualized by staining with 0.5 μg/ml ethidium bromide.

### Cloning and sequencing of *Ld-RAC/AKT-*like gene

The cDNA generated as above was submitted to PCR by using the following sense and antisense primers: 5′-CAC CAT GAG TGG TTA TTT GAA GGT GCT-3′ and 5′-GGA TCC CTA CTT CGT GGG CTT GTC G-3′, designed from *L. major* LmjF.30.0800 (GenBank/EMBL accession no. XM_001684621). PCR reaction was performed in the presence of 8% DMSO, 1.5 mM MgCl_2_, 200 μM dNTPs, 2.5 units of Go*Taq* DNA polymerase, and 1.5 units Pfu DNA polymerase (Promega). PCR was performed in a GeneAmp PCR system model 9600 (Perkin-Elmer). PCR amplification was as follows: 1 cycle at 95 °C for 5 min as an initial denaturation step, then denaturation at 95 °C for 30 s, annealing for 50 s at 61 °C, and extension at 72 °C for 1 min (35 cycles), followed by further incubation for 15 min at 72 °C (1 cycle). PCR product (about 1.5 Kb) was electrophoresed on 1% agarose gels in 1× TAE buffer (40 mM Tris-acetate, 1 mM EDTA (pH 8.0)), and visualized by ethidium bromide staining. PCR bands were cut, and the amplified DNA was isolated by using the GFX PCR DNA and Gel Band Purification kit (GE Healthcare), following the manufacturer’s instructions. Quantification of the isolated DNA was performed by using a NanoDrop™ 8000 spectrophotometer (Thermo Scientific, Waltham, MA, USA). The 1533-bp amplified product was cloned into the PCR 2.1 TOPO® vector (Invitrogen, Carlsbad, CA, USA), following the manufacturer’s instructions, and using a vector/insert ratio of 1:3. DNA sequencing was performed by thermal cycle sequencing using BigDye Terminator v3.1 Cycle Sequencing kit (Applied Biosystems, Foster City, CA, USA). DNA sequencing was performed on both strands from at least 6 independent cDNA clones.

### Bioinformatic analysis of protein sequence identity and similarity

BLASTX, ClustalW2, PROSITE and UniProt databases were used to determine percentages of identity and similarity among different proteins and domains. The UniProt accession numbers used in sequence analysis were as follows: *H. sapiens AKT1* (P31749), *H. sapiens AKT2* (P31751), *H. sapiens AKT3* (Q9Y243), *M. musculus* (P31748), *C. familiaris* (E2RJX4), *D. melanogaster* (Q8INB9), *S. mansoni* (G4M056), *C. elegans* (Q17941), *D. discoideum* (P54644), *E. histolytica* (Q761W9), *L. mexicana* (E9B0K7), *L. braziliensis* (A4HI35), *L. infantum* (A4I5B1), *L. major* (Q4Q7M5), *L. donovani* (Nepal) (E9BLH8), *L. donovani* (India) (S6CXR4) (this work), *L. panamensis* (A0A0F6QP47), *T. cruzi* (Q4D6D3), *T. brucei* (Q584T1), *T. vivax* (G0TWP8), *T. rubrum* (F2SV36), *G. intestinalis* (C6M0B9), *P. yoelii* (Q7RSF6), *P. falciparum* (Q8I4W3).

### Protein modeling and inhibitor docking

Because the 3D structure of the *Ld*-RAC/AKT-like protein has not been resolved experimentally, we used a virtual structural model based on its homology with human AKT1 protein, previously reported in the Protein Data Bank (PDB). The model was built using the ESyPred3D Web Server 1.0 program [30]. The structure of the AKT inhibitor X was obtained from ZINC database [31] in mol2 format. To improve the modeling of the putative interaction between protein and ligand, we carried out a pairwise alignment between *Ld*-RAC/AKT-like protein and human AKT1, mapping the residues of the active site [32]. Then, the inhibitor was docked into the *Ld*-RAC/AKT-like protein using the AutoDock VINA software [33].

### Statistical analysis

The data given are the mean values (± standard deviation, SD) of the indicated number of experiments. Statistical significance was determined by a Student’s *t*-test. Differences were considered significant at a *P*-value of < 0.05.

## Results

### *Leishmania* parasites express an AKT-immunoreactive protein that is upregulated under stress conditions

**Fig. 1:**
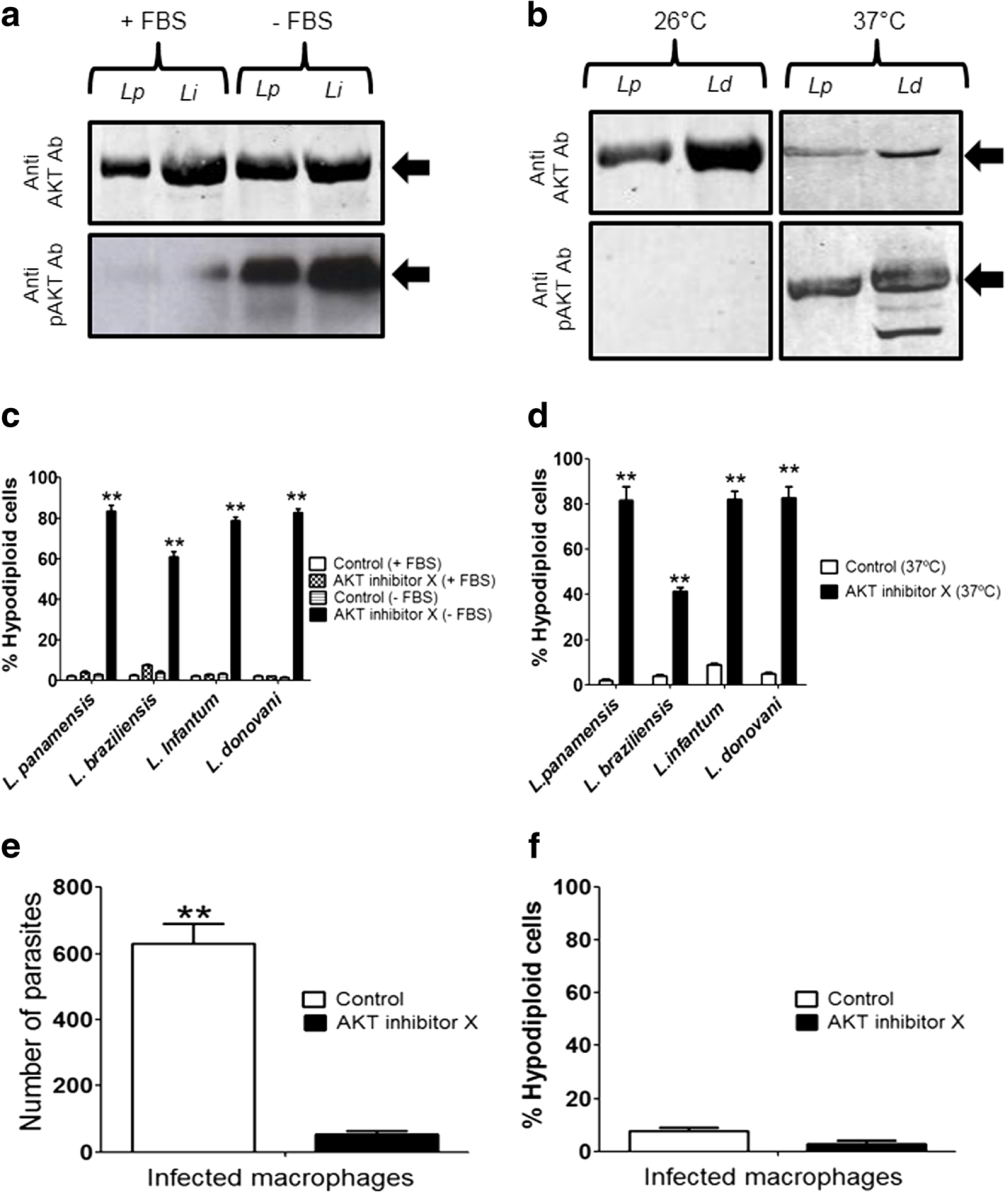
Anti-AKT and anti-pAKT immunoreactive bands in *Leishmania* parasites and *Leishmania* killing by AKT inhibitor X. **a** *L. panamensis* (*Lp*) and *L. infantum* (*Li*) promastigotes were cultured in the presence (+FBS) or absence (−FBS) of FBS, and protein extracts were submitted to Western blot using anti-human AKT1/2/3 and anti-pAKT(Thr308) polyclonal antibodies. **b** *L. panamensis* (*Lp*) and *L. donovani* (*Ld*) promastigotes were cultured at 26 °C or 37 °C, and protein extracts were submitted to Western blot using anti-human AKT1/2/3 and anti-pAKT(Thr308) polyclonal antibodies. The positions of the immunoreactive bands are indicated by arrows. **c** Promastigotes of different *Leishmania* spp. were incubated for 14 h at 26 °C in the presence (+FBS) or absence (−FBS) of FBS, and without (Control) or with 5 μM AKT inhibitor X. Then, parasites were collected and analyzed for the induction of apoptosis-like cell death as assessed by the percentage of parasites in the sub-G_0_/G_1_ region (hypodiploid cells) by flow cytometry. **d** Promastigotes of different *Leishmania* spp. were incubated at 37 °C for 3 h in the presence or absence (Control) of 5 μM AKT inhibitor X. Then, parasites were collected and analyzed for the induction of apoptosis-like cell death as assessed by the percentage of parasites in the sub-G_0_/G_1_ region (hypodiploid cells) by flow cytometry. **e**, **f** J774 macrophage-like cells infected with *L. panamensis* were untreated (Control) or treated with 10 μM AKT inhibitor X for 24 h. Then, the number of intracellular amastigotes was quantified by Giemsa staining (**e**), and the percentage of apoptotic macrophages was assessed through the percentage of mammalian cells at the sub-G_0_/G_1_ region (hypodiploid cells) by flow cytometry (**f**). Data shown are means ± SD or representative of 3 independent experiments. Asterisks indicate statistically significant differences with respect to control values (**P < 0.01). Western blot experiments shown are representative of 3 performed

Because AKT signaling plays a critical role in the survival of mammalian cells, we examined whether anti-human AKT antibodies recognized a band in *Leishmania* parasites. We found that Western blot analyses with anti-human AKT polyclonal antibody of *L. panamensis* (MHOM/CO/87/UA140) and *L. infantum* (MCAN/ES/96/BCN150) promastigotes, previously grown in the presence or absence of fetal bovine serum (FBS), recognized a band of about 57 kDa (Fig. 1a). Interestingly, we also found a 57 kDa band that was immunoreactive against anti-human pAKT(Thr308) antibodies, highly upregulated when parasites were incubated in the absence of serum (Fig. 1a). Similarly, *L. panamensis* and *L. donovani* (MHOM/IN/80/Dd8) promastigotes also expressed the above AKT-immunoreactive protein (Fig. 1b), as well as a protein of about 57 kDa by using the anti-human pAKT(Thr308) antibody when parasites were submitted to a heat shock at 37 °C (Fig. 1b). However, no band was detected when anti-human pAKT(Ser473) was used in parasites grown under stressful conditions. These data suggest that *Leishmania* spp. promastigotes express an AKT-like protein that could be recognized by the anti-human AKT antibody, and that this parasite protein might be phosphorylated in Thr residues.

### AKT inhibition induces apoptosis-like cell death in *Leishmania* promastigotes and amastigotes

Next, we analyzed the effect of AKT inhibitor X that prevents phosphorylation of AKT in mammalian cells [34]. We found that incubation of 2 × 10^6^ *Leishmania* promastigotes from different species, including *L. panamensis*, *L. braziliensis*, *L. infantum* and *L. donovani*, with AKT inhibitor X did not affect cell viability, provided serum was present in the culture medium (Fig. 1c), but AKT inhibitor X induced a potent apoptosis-like cell death, determined by an increase in the percentage of hypodiploid cells (sub-G_0_/G_1_ region in cell cycle analysis), as an indication of DNA breakdown, when parasites were submitted to stress conditions, such as serum deprivation or incubation at 37 °C (Fig. 1c, d). Moreover, we also found that AKT inhibitor X killed J774 macrophage-residing *L. panamensis* amastigotes, as assessed by a dramatic decrease in the parasitic burden of macrophages following Giemsa staining (Fig. 1e), whereas macrophages were spared as assessed by no DNA breakdown detection in macrophages analyzed by flow cytometry (Fig. 1f).

### Cloning and sequence of a gene coding for a protein in *Leishmania donovani* (MHOM/IN/80Dd8) that has similarities to mammalian AKT

**Fig. 2:**
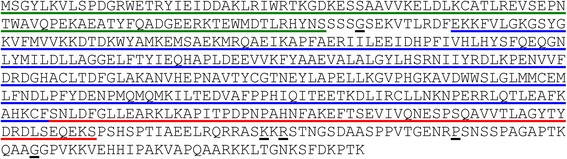
Amino acid sequence of *Ld*-RAC/AKT-like protein from *L. donovani*. The nucleotide and predicted amino acid sequences of *Ld-RAC/AKT-*like gene are available from GenBank/EMBL database under accession number HF548848. The PH, kinase and AGC kinase domains of the *Ld*-RAC/AKT-like protein are underlined in green, blue and red, respectively. This primary sequence shows five differences in amino acids (underlined in black) when compared to a previously homologous sequence from another *L. donovani* strain (MHOM/NP/02/BPK282A1) (Genbank/EMBL accession number: XM_003862750)

Because the above data suggested the presence of an AKT-like protein in *Leishmania* parasites, we analyzed the presence of a putative sequence similar to mammalian AKT in the already sequenced genome of Nepalese *L. donovani* (MHOM/NP/02/BPK282A1). We found that a gene named as *LDBPK_300850* (RAC serine-threonine kinase, putative) showed a 26.3% identity with the sequence of human AKT1. By using specific primers designed from *LDBPK_300850*, we generated RT-PCR fragments from Indian *L. donovani* (MHOM/IN/80Dd8) mRNA, which were subsequently cloned and sequenced. Following this strategy, we isolated and sequenced the *LDBPK_300850* homologue from Indian *L. donovani* (MHOM/IN/80Dd8). We coined this gene as *Ld-RAC/AKT-*like gene, indicating its origin and the similarity to the previous parasite *RAC*-like and mammalian *AKT* genes. The *Ld*-*RAC*/*AKT-*like cDNA codes for a protein sequence of 510 amino acids with a deduced molecular mass of about 57,612.5 Da and a theoretical isoelectric point (pI) of 6.25. This *Ld*-RAC/AKT-like protein contains the typical pleckstrin (PH domain), protein kinase and AGC kinase domains, which represent the major hallmarks of mammalian PKB/AKT proteins (Fig. 2).

We found a small number of differences, namely 32 nucleotides (97.8% identity) and 5 amino acid residues (99% identity), when the corresponding Indian *L. donovani* (MHOM/IN/80Dd8) and Nepalese *L. donovani* (MHOM/NP/02/BPK282A1) homologous sequences were compared (Fig. 2 and Table 1). The amino acid sequence of *Ld*-RAC/AKT-like protein showed a 26.5% identity and 43.2% similarity to the corresponding human AKT1 coding region (Table 1). Using the ESyPred3D Web Server 1.0 program, we found that the structure of this *Ld*-RAC/AKT-like protein is similar to that of human AKT1 (Additional file 1: Figure S1a). Using the Docking Software AutoDock VINA, we found that the *Ld*-RAC/AKT-like protein probably interacts with AKT inhibitor X (Additional file 1: Figure S1b). Using the AutoDock VINA software for binding prediction, the AKT inhibitor X docked into the *Ld*-RAD/AKT-like protein with a -7.5 kcal/mol, whereas the interaction between AKT inhibitor X and human AKT1 was -8.8 kcal/mol, in a 0 to -14 kcal/mol scale, the latter value representing the highest probability of interaction.

**Table 1:** Comparison of *Ld-RAC/AKT-*like gene with trypanosomatid and human homologues. Identity and similarity percentages of the full cDNA and protein sequences are shown as compared to the herein reported *Ld-RAC/AKT*-like gene from *L*. *donovani* (MHOM/IN/80/Dd8)

Species	cDNA % identity	Protein % identity	Protein % similarity
*L. donovani (Nepal)*	97.8	98.0	99.2
*L. braziliensis*	89.6	92.9	94.5
*L. infantum*	97.7	98.8	99.2
*L. major*	98.0	98.8	98.8
*L. mexicana*	96.0	96.7	97.6
*L. panamensis*	90.1	93.9	96.6
*T. cruzi*	45.6	32.5	46.3
*T. vivax*	41.8	32.9	48.1
*T. brucei*	42.0	34.6	48.4
*Human AKT1*	43.9	26.5	43.2
*Human AKT2*	42.1	26.1	41.5
*Human AKT3*	43.8	25.4	40.7

### Presence of AKT-like genes in *Leishmania* and *Trypanosoma* parasites

**Fig. 3:**
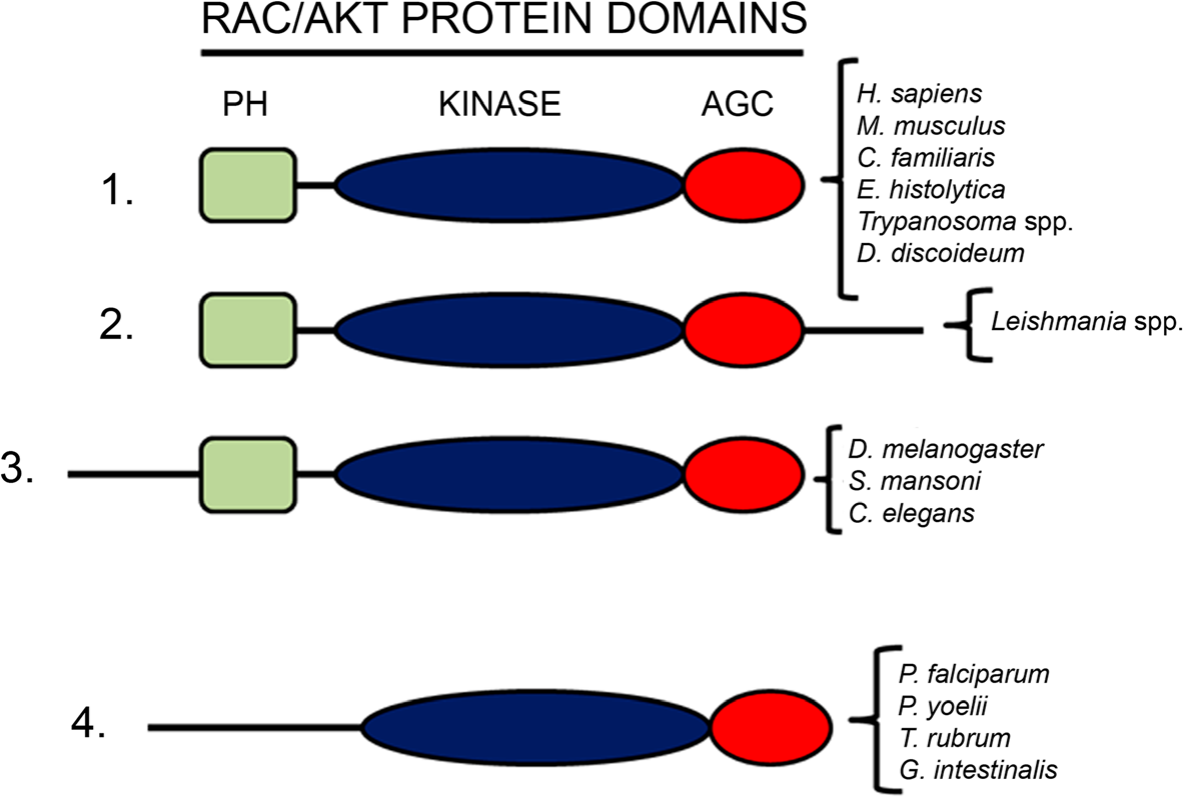
Classification of RAC/AKT-like proteins in four major subcategories. This scheme depicts the alignment of the different amino acid sequences corresponding to RAC/AKT-like proteins in four major subcategories, based on the presence of the three typical PH (green), kinase (blue) and AGC (red) domains of AKT proteins. The last group does not contain the PH domain, but it is included here because of its homology regarding the other two domains. The *Leishmania* group shows a longer C-terminal sequence

A comparison of the *Ld*-RAC/AKT-like protein sequence with those derived from the genome sequences already available in the databases for *Leishmania* and *Trypanosoma* parasites showed a strong similarity in their respective primary structures (*Leishmania*: 92.9–99% identity, 94.5–99.2% similarity; *Trypanosoma*: 32.5–34.6% identity, 46.3–48.4% similarity) (Table 1). This protein also contained the 12 conserved subdomains of the eukaryotic protein kinase domain, which were also present in the predicted homologous sequences of trypanosomatids (Additional file 2: Figure S2). More extensive comparative analyses of the sequences showing homology with human AKT in different biological systems, regarding whole sequences as well as the major PH, kinase and AGC domains, are shown in Table 2 and Additional file 3: Figure S3, and this comparative analysis prompted us to classify this large family of genes in four major subcategories as shown in Fig. 3. The *Leishmania* group shows a longer C-terminal sequence, whereas there is another subcategory that lacks the PH domain (Fig. 3).

**Table 2:** Comparison of human AKT1 with RAC/AKT kinases from different origins. Identity and similarity percentages of the full protein as well as percentages of similarity in the distinct domains are shown. Sequence alignment was performed using the Needleman-Wunsch algorithm

Species	Identity	Similarity			
	Full Protein	Full Protein	PH Domain	Kinase Domain	AGC Domain
*H. sapiens*	100	100	100	100	100
*M. musculus*	98.3	99.0	99.0	99.6	97.2
*C. familiaris*	96.9	98.3	100	99.2	93.1
*D. melanogaster*	48.6	60.5	71.3	88.4	62.3
*S. mansoni*	47.9	58.5	74.0	81.5	52.6
*C. elegans*	51.8	65.8	72.1	83.0	60.3
*D. discoideum*	41.5	58.5	43.9	72.7	46.7
*E. histolytica*	37.5	56.0	45.8	70.3	41.8
*L. mexicana*	26.3	43.0	28.9	61.7	36.8
*L. braziliensis*	26.8	42.1	28.7	61.7	21.7
*L. infantum*	26.3	42.8	29.8	61.7	29.6
*L. major*	26.0	41.9	26.0	61.7	29.6
*L. donovani (India)*	26.5	43.2	29.8	61.7	29.6
*L. donovani (Nepal)*	26.3	42.8	29.8	61.7	29.6
*L. panamensis*	35.0	55.0	43.0	63.0	19.3
*T. cruzi*	33.0	52.6	42.0	64.6	37.2
*T. brucei*	32.7	50.9	36.8	63.6	41.0
*T. vivax*	34.7	54.3	42.3	64.8	45.2
*T. rubrum*	25.9	38.2	3.0	69.3	43.4
*G. intestinalis*	27.2	41.1	14.9	58.0	38.4
*P. yoelii*	23.2	35.0	3.9	71.8	50.0
*P. falciparum*	23.1	34.8	6.7	74.5	45.3

### Differential presence of Thr and Ser amino acid residues at the phosphorylation sites in mammalian and *Leishmania* AKT-like genes

**Fig. 4:**
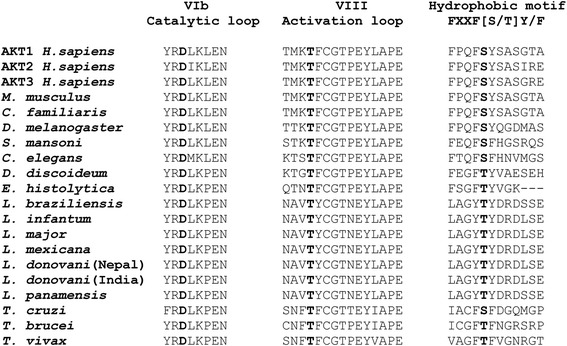
Differences in the amino acids involved in AKT phosphorylation in the RAC/AKT-like protein family. The alignments of the distinct VIb catalytic loop, VIII activation loop and hydrophobic motif, where D is important for phosphotransference, T for AKT phosphoactivation, and S for AKT activity potentiation, respectively, are shown, with the above amino acid residues highlighted in bold. RAC/AKT-like proteins from *Leishmania* spp. have T instead of S in the hydrophobic motif. Likewise, proteins derived from *T. brucei* and *T. vivax* have T instead of S, whereas *T. cruzi* shows the same S residue as the human AKT1

The full activation of mammalian AKT is accomplished by its phosphorylation at two key residues, Thr308 and Ser473 [13]. The Thr308 site is located in the activation loop within the catalytic domain, while Ser473 is located in the C-terminal hydrophobic motif of mammalian AKT. Interestingly, AKT-like proteins from *Leishmania* spp. have the Thr residue in the activation loop, but showed another Thr residue instead of the mammalian Ser residue at the hydrophobic motif (Fig. 4). Thus, the typical FPQF**S**Y hydrophobic motif of mammalian AKT becomes LAGY**T**Y in *Leishmania* parasites (Fig. 4). The absence of this Ser residue might explain the lack of detection of a protein band in *Leishmania* spp. extracts when an anti-human pAKT(Ser473) antibody was used, as stated above. Likewise, the corresponding AKT-like sequences from *T. brucei* and *T. vivax* also lacked the Ser residue at the hydrophobic motif (Fig. 4). However, the AKT-like amino acid sequence from *T. cruzi* contained the Ser residue at the hydrophobic motif (IACF**S**F) (Fig. 4), and therefore it was the only AKT-like sequence of the Trypanosomatidae family that showed homology with mammalian AKT regarding these critical phosphorylation sites.

## Discussion

Our data revealed that *Leishmania donovani* parasites express the *Ld*-*RAC*/*AKT-*like gene encoding a protein of 57.6 kDa that shows a significant identity (26.5%) and similarity (43.2%) with mammalian AKT1. Antibodies against human AKT recognize a protein in *Leishmania* spp. of about 57 kDa that is suggested to be phosphorylated under stressful conditions, such as nutrient deprivation and heat shock. Comparison of the *Ld*-RAC/AKT-like protein amino acid sequence with those of known eukaryotic Ser/Thr protein kinases indicates that the newly described *L. donovani* protein belongs to the RAC or PKB/AKT subgroup of proteins. Because PKB/AKT has a major prosurvival role in mammalian cells [10, 20], our data suggest that the *Ld*-RAC/AKT-like protein could represent part of a PKB/AKT signaling pathway present in trypanosomatids, and thereby might play a role in *Leishmania* survival during its complex life-cycle. On these grounds, inhibition of this AKT-like signaling pathway in *Leishmania* could be a novel approach to the search of anti-*Leishmania* drugs. The induction of apoptosis-like cell death in *Leishmania* spp. by the AKT inhibitor X, when parasites are submitted to stress conditions, further supports the involvement of an AKT signaling pathway in *Leishmania* parasite survival. It is tempting to suggest that parasites growing in nutrient-deficient medium or at high temperature might defend themselves by triggering phosphorylation of the *Ld*-RAC/AKT-like protein, thus prompting a survival response to detrimental conditions. Our data suggest that blocking this response by an AKT inhibitor might facilitate *Leishmania* cell death. *Ld*-RAC/AKT-like protein and human AKT1 show a 26.5% identity, suggesting the existence of differences that might be exploited to identify drugs inhibiting selectively to the *Leishmania* AKT homologue. In this regard, pleckstrin homology (PH) and AGC domains, which are involved in the membrane localization and function of AKT proteins [35–37], could represent interesting targets for the search of specific antileishmanial drugs, as the percentage of similarity between the amino acid sequences of *Leishmania* parasites with respect to that of human cells ranges 26–43% for the PH domain and from 19.3 to 36.8% for the AGC domain in the distinct *Leishmania* species (Table 2). Thus, the search for specific amino acid sequences as drug targets in these domains of the RAC/AKT-like proteins in *Leishmania* parasites could minimize putative side effects by their absence in the cognate human counterparts.

Following primary sequence comparison, we postulate the evolutionary conservation of the PKB/AKT signaling pathway in different parasites. Trypanosomatidae parasite homologues contain the hallmark traits of PKB/AKT proteins, such as the presence of pleckstrin, protein kinase and AGC kinase domains. Here we classify the RAC/AKT-like family of proteins in four major subcategories that include the parasitic homologues, *Leishmania* parasites forming a major group on their own (Fig. 3). Only one subcategory, comprised of *P. falciparum*, *P. yoelii*, *T. rubrum* and *G. intestinalis* (a.k.a. *Giardia lamblia*), lacks the pleckstrin-homologous domain. Interestingly, while mammalian AKT is activated through phosphorylation at both Thr308 and Ser473 key residues in the activation loop and hydrophobic motif [13], the *Leishmania* AKT homologue lacks the corresponding Ser residue and shows Thr in both motifs. It is worth to note that, unlike *Leishmania* parasites, *T. cruzi* AKT-like homologue, but not *T. brucei* and *T. vivax* homologues, contains both Thr and Ser residues in its sequence, similarly to what occurs in human PKB/AKT (Fig. 4).

Activation of host cell phosphatidylinositol 3-kinase and PKB/AKT activities by *T. cruzi* has been shown to be an early invasion signal required for trypomastigote internalization [38]. Thus, PKB/AKT signaling is suggested to be associated with parasite invasion and survival, and therefore it might represent a novel target for the treatment of leishmaniasis. Taken together, the identification of *Ld*-RAC/AKT-like protein may lead to the eventual design of approaches and drugs targeting *Leishmania* parasites.

## Conclusions

In this study, a new gene, *Ld-RAC/AKT-*like gene, was identified in *Leishmania donovani*, which showed a significant similarity with mammalian PKB/AKT. Anti-human AKT antibodies recognized a band of about 57 kDa in *Leishmania* parasites, which was suggested to be phosphorylated under parasite exposure to stressful conditions, such as nutrient deprivation or heat shock. Incubation of AKT inhibitor X with *Leishmania* spp. promastigotes under stressful conditions or with *Leishmania*-infected macrophages led to parasite cell death. Following extensive comparative protein sequence analyses in different biological systems, regarding whole sequences, as well as the major PH, kinase and AGC domains, RAC/AKT proteins could be classified into four major groups, with those derived from *Leishmania* spp. constituting a subgroup with unique sequence features within the general forming AKT-like protein family. Taken together, these data suggest that the new *Ld-RAC/AKT-*like gene herein cloned represents a *Leishmania* orthologue of mammalian AKT involved in parasite stress response and survival, and therefore could become a novel therapeutic and druggable target in leishmaniasis therapy.

## Additional files

Additional file 1: Figure S1. Prediction of 3D structures of human AKT1 and *Ld*-RAC/AKT-like protein, and interaction with AKT inhibitor X. **a** Human AKT1 and *Ld*-RAC/AKT-like proteins were modeled using the ESyPred3D Web Server 1.0 program. The α-helical and β-strand domains are colored magenta and yellow, respectively, while turns are colored violet. **b** Different views of the predicted interaction between *Ld*-RAC/AKT-like protein and AKT inhibitor X using the AutoDock VINA software. (upper left) The whole protein surface is shown and the black arrow indicates the location of the inhibitor X. (upper right) The cavity of the ATP binding pocket at higher magnification is displayed, showing the interaction with AKT inhibitor X. The images at the lower panels show the inhibitor X within the ATP binding pocket in vertical and horizontal position interacting with the protein (TIFF 9524 kb)

Additional file 2: Figure S2. Alignment of the catalytic domains of the distinct RAC/AKT-like proteins present in *Leishmania* spp. and *Trypanosoma* spp. parasites. Multiple sequence alignment was performed using the ClustalW program. The accession numbers of the UniProt sequences analyzed here are as follows: *L. braziliensis* (A4HI35), *L. panamensis* (A0A0F6QP47), *L. mexicana* (E9B0K7), *L. major* (Q4Q7M5), *L. donovani* (India) (S6CXR4) (this work), *L. donovani* (Nepal) (E9BLH8), *L. infantum* (A4I5B1), *T. cruzi* (Q4D6D3), *T. brucei* (Q584T1), *T. vivax* (G0TWP8). The distinct conserved subdomains of the catalytic domain are indicated by Roman numerals (I-XI). Asterisks indicate identity and small dots represent similarity (DOCX 18 kb)

Additional file 3: Figure S3. Analysis of RAC/AKT proteins from different origins. Multiple sequence alignment was performed using the Clustal O (1.2.4) program. Asterisks indicate identity and small dots represent similarity (DOC 50 kb)

## Abbreviations

FACS: fluorescence-activated cell sorting
FBS: fetal bovine serum
GSK-3: glycogen synthase kinase-3
PBS: phosphate-buffered saline
PDB: Protein Data Bank
PH: pleckstrin homology domain
pI: isoelectric point
PKB: protein kinase B
RAC: related to A and C protein kinases

## References

[1] WHO. Control of the leishmaniases. World Health Organ Tech Rep Ser. 2010;949:1–186. 21485694

[2] Ivens AC, Peacock CS, Worthey EA, Murphy L, Aggarwal G, Berriman M, et al.The genome of the kinetoplastid parasite, *Leishmania major*. Science. 2005;309:436–42. 10.1126/science.1112680 PMC1470643 16020728

[3] Peacock CS, Seeger K, Harris D, Murphy L, Ruiz JC, Quail MA (2007). Comparative genomic analysis of three *Leishmania* species that cause diverse human disease. Nat Genet.

[4] Downing T, Imamura H, Decuypere S, Clark TG, Coombs GH, Cotton JA (2011). Whole genome sequencing of multiple *Leishmania donovani* clinical isolates provides insights into population structure and mechanisms of drug resistance. Genome Res.

[5] Rogers MB, Hilley JD, Dickens NJ, Wilkes J, Bates PA, Depledge DP (2011). Chromosome and gene copy number variation allow major structural change between species and strains of *Leishmania*. Genome Res.

[6] Real F, Vidal RO, Carazzolle MF, Mondego JM, Costa GG, Herai RH (2013). The genome sequence of *Leishmania* (*Leishmania*) *amazonensis*: functional annotation and extended analysis of gene models. DNA Res.

[7] Llanes A, Restrepo CM, Del Vecchio G, Anguizola FJ, Lleonart R (2015). The genome of *Leishmania panamensis*: insights into genomics of the *L.* (*Viannia*) subgenus. Sci Rep.

[8] LoPiccolo J, Granville CA, Gills JJ, Dennis PA (2007). Targeting Akt in cancer therapy. Anti-Cancer Drugs.

[9] Falasca M (2010). PI3K/Akt signalling pathway specific inhibitors: a novel strategy to sensitize cancer cells to anti-cancer drugs. Curr Pharm Des.

[10] Gdowski A, Panchoo M, Treuren TV, Basu A (2016). Emerging therapeutics for targeting Akt in cancer. Front Biosci (Landmark Ed).

[11] Asati V, Mahapatra DK, Bharti SK (2016). PI3K/Akt/mTOR and Ras/Raf/MEK/ERK signaling pathways inhibitors as anticancer agents: structural and pharmacological perspectives. Eur J Med Chem.

[12] Brazil DP, Hemmings BA (2001). Ten years of protein kinase B signalling: a hard Akt to follow. Trends Biochem Sci.

[13] Franke TF (2008). PI3K/Akt: getting it right matters. Oncogene.

[14] LoPiccolo J, Blumenthal GM, Bernstein WB, Dennis PA (2008). Targeting the PI3K/Akt/mTOR pathway: effective combinations and clinical considerations. Drug Resist Updat.

[15] Hers I, Vincent EE, Tavare JM (2011). Akt signalling in health and disease. Cell Signal.

[16] Ojo KK, Gillespie JR, Riechers AJ, Napuli AJ, Verlinde CL, Buckner FS (2008). Glycogen synthase kinase 3 is a potential drug target for African trypanosomiasis therapy. Antimicrob Agents Chemother.

[17] Xingi E, Smirlis D, Myrianthopoulos V, Magiatis P, Grant KM, Meijer L (2009). 6-Br-5methylindirubin-3'oxime (5-Me-6-BIO) targeting the leishmanial glycogen synthase kinase-3 (GSK-3) short form affects cell-cycle progression and induces apoptosis-like death: exploitation of GSK-3 for treating leishmaniasis. Int J Parasitol.

[18] Ojo KK, Arakaki TL, Napuli AJ, Inampudi KK, Keyloun KR, Zhang L (2011). Structure determination of glycogen synthase kinase-3 from *Leishmania major* and comparative inhibitor structure-activity relationships with *Trypanosoma brucei* GSK-3. Mol Biochem Parasitol.

[19] Liang J, Slingerland JM (2003). Multiple roles of the PI3K/PKB (Akt) pathway in cell cycle progression. Cell Cycle.

[20] Song G, Ouyang G, Bao S (2005). The activation of Akt/PKB signaling pathway and cell survival. J Cell Mol Med.

[21] Scanga SE, Ruel L, Binari RC, Snow B, Stambolic V, Bouchard D (2000). The conserved PI3'K/PTEN/Akt signaling pathway regulates both cell size and survival in *Drosophila*. Oncogene.

[22] Paradis S, Ruvkun G (1998). *Caenorhabditis elegans* Akt/PKB transduces insulin receptor-like signals from AGE-1 PI3 kinase to the DAF-16 transcription factor. Genes Dev.

[23] Meili R, Ellsworth C, Firtel RA (2000). A novel Akt/PKB-related kinase is essential for morphogenesis in *Dictyostelium*. Curr Biol.

[24] Que X, Reed SL (1994). Expression and characterization of a *rac* family protein kinase of *Entamoeba histolytica*. Mol Biochem Parasitol.

[25] Kim KT, Mok MT, Edwards MR (2005). Protein kinase B from *Giardia intestinalis*. Biochem Biophys Res Commun.

[26] Pascuccelli V, Labriola C, Tellez-Inon MT, Parodi AJ (1999). Molecular and biochemical characterization of a protein kinase B from *Trypanosoma cruzi*. Mol Biochem Parasitol.

[27] Gajate C, Santos-Beneit AM, Macho A, Lazaro M, Hernandez-De Rojas A, Modolell M, Munoz E, Mollinedo F (2000). Involvement of mitochondria and caspase-3 in ET-18-OCH_3_-induced apoptosis of human leukemic cells. Int J Cancer.

[28] Gajate C, Barasoain I, Andreu JM, Mollinedo F (2000). Induction of apoptosis in leukemic cells by the reversible microtubule-disrupting agent 2-methoxy-5-(2′,3′,4′-trimethoxyphenyl)-2,4,6-cycloheptatrien-1-one: protection by Bcl-2 and Bcl-X_L_ and cell cycle arrest. Cancer Res.

[29] Ramirez JR, Berberich C, Jaramillo A, Alonso C, Velez IV (1998). Molecular and antigenic characterization of the *Leishmania* (*Viannia*) *panamensis* kinetoplastid membrane protein-11. Mem Inst Oswaldo Cruz.

[30] Lambert C, Leonard N, De Bolle X, Depiereux E (2002). ESyPred3D: prediction of proteins 3D structures. Bioinformatics.

[31] Irwin JJ, Sterling T, Mysinger MM, Bolstad ES, Coleman RG. ZINC: a free tool to discover chemistry for biology. J Chem Inf Model. 2012;52:1757–68. 10.1021/ci3001277 PMC3402020 22587354

[32] Blake JD, Cohen FE (2001). Pairwise sequence alignment below the twilight zone. J Mol Biol.

[33] Trott O, Olson AJ (2010). AutoDock Vina: improving the speed and accuracy of docking with a new scoring function, efficient optimization, and multithreading. J Comput Chem.

[34] Thimmaiah KN, Easton JB, Germain GS, Morton CL, Kamath S, Buolamwini JK, Houghton PJ (2005). Identification of N10-substituted phenoxazines as potent and specific inhibitors of Akt signaling. J Biol Chem.

[35] Scheffzek K, Welti S (2012). Pleckstrin homology (PH) like domains - versatile modules in protein-protein interaction platforms. FEBS Lett.

[36] Pearce LR, Komander D, Alessi DR (2010). The nuts and bolts of AGC protein kinases. Nat Rev Mol Cell Biol.

[37] Arencibia JM, Pastor-Flores D, Bauer AF, Schulze JO, Biondi RM (1834). AGC protein kinases: from structural mechanism of regulation to allosteric drug development for the treatment of human diseases. Biochim Biophys Acta.

[38] Wilkowsky SE, Barbieri MA, Stahl P, Isola EL (2001). *Trypanosoma cruzi*: phosphatidylinositol 3-kinase and protein kinase B activation is associated with parasite invasion. Exp Cell Res.

