# Supplementary figures and images for: Identification of a RAC/AKT-like gene in Leishmania parasites as a putative therapeutic target in leishmaniasis
Source: Parasit Vectors. 2017 Oct 10;10:458. doi: 10.1186/s13071-017-2379-y (PMC5633885; doi:10.1186/s13071-017-2379-y)

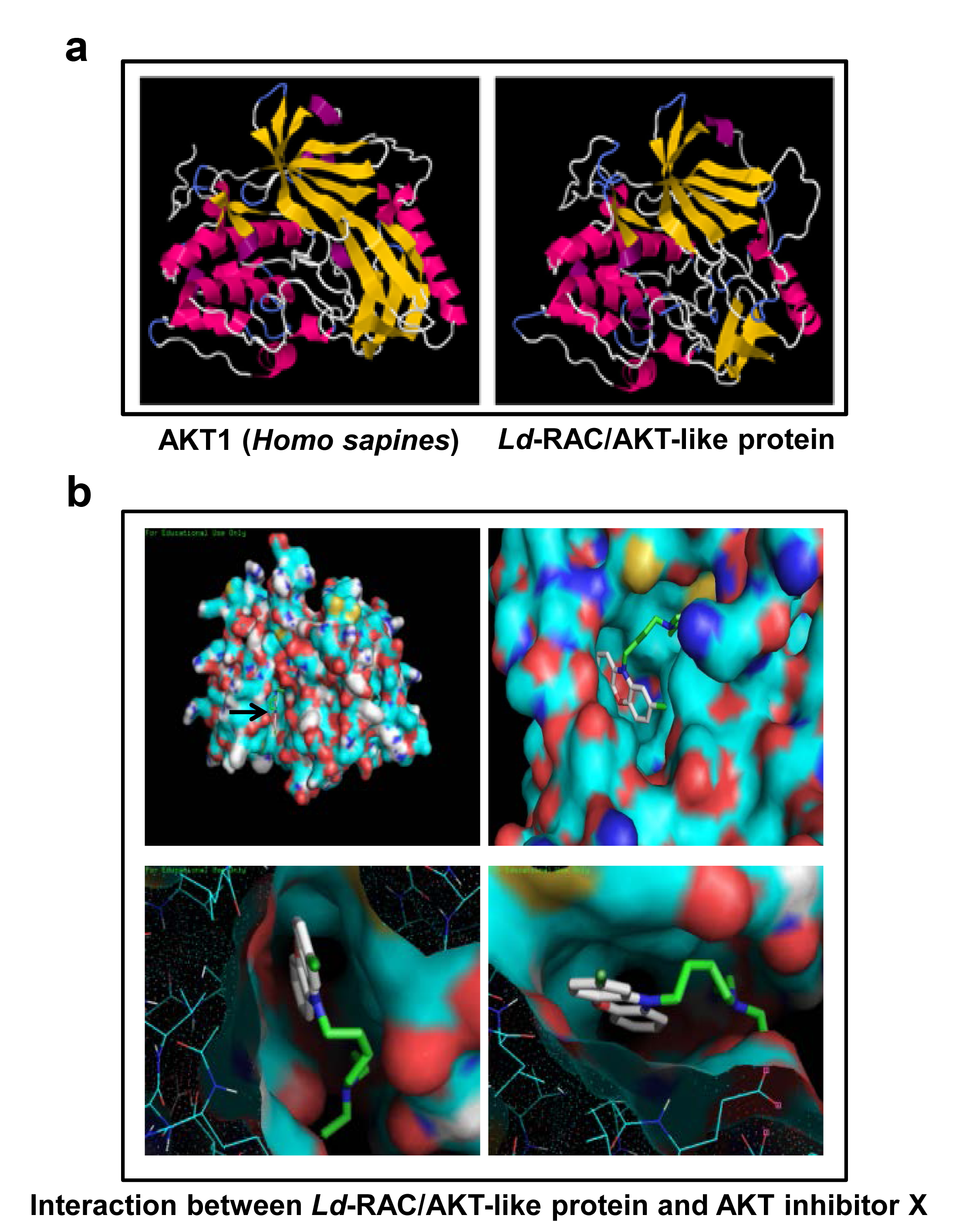

Supplement: Supplementary file 1 — Prediction of 3D structures of human AKT1 and Ld-RAC/AKT-like protein, and interaction with AKT inhibitor X. a Human AKT1 and Ld-RAC/AKT-like proteins were modeled using the ESyPred3D Web Server 1.0 program. The α-helical and β-strand domains are colored magenta and yellow, respectively, while turns are colored violet. b Different views of the predicted interaction between Ld-RAC/AKT-like protein and AKT inhibitor X using the AutoDock VINA software. (upper left) The whole protein surface is shown and the black arrow indicates the location of the inhibitor X. (upper right) The cavity of the ATP binding pocket at higher magnification is displayed, showing the interaction with AKT inhibitor X. The images at the lower panels show the inhibitor X within the ATP binding pocket in vertical and horizontal position interacting with the protein (TIFF 9524 kb) [file 13071_2017_2379_MOESM1_ESM.tif]
