# Supplementary material for: Identification of a RAC/AKT-like gene in Leishmania parasites as a putative therapeutic target in leishmaniasis
Source: Parasit Vectors. 2017 Oct 10;10:458. doi: 10.1186/s13071-017-2379-y (PMC5633885; doi:10.1186/s13071-017-2379-y)
Supplement: Supplementary file 2 — Alignment of the catalytic domains of the distinct RAC/AKT-like proteins present in Leishmania spp. and Trypanosoma spp. parasites. Multiple sequence alignment was performed using the ClustalW program. The accession numbers of the UniProt sequences analyzed here are as follows: L. braziliensis (A4HI35), L. panamensis (A0A0F6QP47), L. mexicana (E9B0K7), L. major (Q4Q7M5), L. donovani (India) (S6CXR4) (this work), L. donovani (Nepal) (E9BLH8), L. infantum (A4I5B1), T. cruzi (Q4D6D3), T. brucei (Q584T1), T. vivax (G0TWP8). The distinct conserved subdomains of the catalytic domain are indicated by Roman numerals (I-XI). Asterisks indicate identity and small dots represent similarity (DOCX 18 kb) [file 13071_2017_2379_MOESM2_ESM.docx]

I II

_____________________ ________________________ ____

*L. braziliensis* --SSIGSEKVTLSDFEKKFVLGKGSYGKVFMVVKKDTDKWYAMKEMSAEKMRQAE-IKAP

*L. panamensis* --SNIGSEKVTLRDFEKKFVLGKGSYGKVFMVVKKDTDKWYAMKEMSAEKMRQAE-IKAP

*L. mexicana* --GSTGSEKVTLRDFEKKFVLGKGSYGKVFMVVKKDTDKWYAMKEMSAEKMRQAE-IKAP

*L. major* --SSSGSEKVTLRDFEKKFVLGKGSYGKVFMVVKKDTDKWYAMKEMSAEKMRQAE-IKAP

*L. donovani*(I) --SSSGSEKVTLRDFEKKFVLGKGSYGKVFMVVKKDTDKWYAMKEMSAEKMRQAE-IKAP

*L. donovani*(N) --SSSASEKVTLRDFEKKFVLGKGSYGKVFMVVKKDTDKWYAMKEMSAEKMRQAE-IKAP

*L. infantum* --SSSASEKVTLRDFEKKFVLGKGSYGKVFMVVKKDTDKWYAMKEMSAEKMRQAE-IKAP

*T. cruzi* TLYTGGRHKVSLDDFELKATIGAGSFSNVFVAREKSTDKVYAIKEMGKELIQQHNMLSNI

*T. brucei* PLCSGGSNRVSLNDFQFAAKIGKGSFSSVYAATEKATGKTYAIKKMEKEVIERYNMIDNI

*T. vivax* TL--YGDDKVSLNDFEVTTTIGKGSFSFVYAAREKSTNKLYAVKEMKKEVIERANMLENI

. .:*:* **: :* **:. *: . :* *.* **:*:* * :.: : :.

III IV V VIa

__________ ____________ _____________ ________

*L. braziliensis* FAERIILEEIDHPFIVHLHYSFQEQGNLYMILDLLAGGELFTYIEQHAPLDEEVVKFYAA

*L. panamensis* FAERIILEEIDHPFIVHLHYSFQEQGNLYMILDLLAGGELFTYIEQHAPLDEEVVKFYAA

*L. mexicana* FAERIILEEIDHPFIVHLHYSFQEQGNLYMILDLLAGGELFTYIEQHAPLDEEVVKFYAA

*L. major* FAERIILEEIDHPFIVHLHYSFQEQGNLYMILDLLAGGELFTYIEQHAPLDEEVVKFYAA

*L. donovani*(I) FAERIILEEIDHPFIVHLHYSFQEQGNLYMILDLLAGGELFTYIEQHAPLDEEVVKFYAA

*L. donovani*(N) FAERIILEEIDHPFIVHLHYSFQEQGNLYMILDLLAGGELFTYIEQHAPLDEEVVKFYAA

*L. infantum* FAERIILEEIDHPFIVHLHYSFQEQGNLYMILDLLAGGELFTYIEQHAPLDEEVVKFYAA

*T. cruzi* AAEKHILQTISHPFIVSLHYAFETKKCLYLVLDFLPGGELFFHLAKEKVFDEYRAKFYCG

*T. brucei* SAERLILQKIDHPFIVSLHYAFQTKGSLYLVMDFLSGGELFFHLESVSVFDEWRAKFYCG

*T. vivax* FTEKRILQDIRHPFIVSLHYAFQTPNCLYLVMDFLPGGELFFHLGNVKTFDEQRAKFYCG

:*: **: * ***** ***:*: **:::*:* ***** :: . :** .***..

VIb VII VIII

____________ ________________ ________________ _____________

*L. braziliensis* EVALALGYLHSRNIIYRDLKPENVVFDHEGHACLTDFGLAKANVHEPNAVTYCGTNEYLA

*L. panamensis* EVALALGYLHSRNIIYRDLKPENVVFDHEGHACLTDFGLAKANVHEPNAVTYCGTNEYLA

*L. mexicana* EVALALGYLHSRNIIYRDLKPENVVFDRDGHACLTDFGLAKANVHEPNAVTYCGTNEYLA

*L. major* EVALALGYLHSRNIIYRDLKPENVVFDRDGHACLTDFGLAKANVHEPNAVTYCGTNEYLA

*L. donovani*(I) EVALALGYLHSRNIIYRDLKPENVVFDRDGHACLTDFGLAKANVHEPNAVTYCGTNEYLA

*L. donovani*(N) EVALALGYLHSRNIIYRDLKPENVVFDRDGHACLTDFGLAKANVHEPNAVTYCGTNEYLA

*L. infantum* EVALALGYLHSRNIIYRDLKPENVVFDRDGHACLTDFGLAKANVHEPNAVTYCGTNEYLA

*T. cruzi* EIALAIGYLHSLDIIFRDLKPENIVLDEDGHACLTDFGLAKMNVSNASNFTFCGTTEYIA

*T. brucei* EIALALGYLHAQDIIYRDLKPENAVLDADGHVCLTDFGLAKMDVRDACNFTFCGTPEYIA

*T. vivax* EIALALEYLHEHNIIYRDLKPENAVLDADGHVCLTDFGLAKMVVSDASNFTFCGTPEYVA

*:***: *** :**:******* *:* :**.********* * : .*:*** **:*

IX X

__ ______________________________ __________________

*L. braziliensis* PELLKGVPHGKAVDWWSLGLMMCEMLFNDLPFYDENPMQMQMKILTEDVSFPS-------

*L. panamensis* PELLKGVPHGKAVDWWSLGLMMCEMLFNDLPFYDENPMQMQMKILTEDVAFPS-------

*L. mexicana* PELLKGVPHGKAVDWWSLGLMMCEMLFNDLPFYDENPMQMQMKILTEDVAFPP-------

*L. major* PELLKGVPHGKAVDWWSLGLMMCEMLFNDLPFYDENPMQMQMKILTEDVAFPP-------

*L. donovani*(I) PELLKGVPHGKAVDWWSLGLMMCEMLFNDLPFYDENPMQMQMKILTEDVAFPP-------

*L. donovani*(N) PELLKGVPHGKAVDWWSLGLMMCEMLFNDLPFYDENPMQMQMKILTEDVAFPP-------

*L. infantum* PELLKGVPHGKAVDWWSLGLMMCEMLFNDLPFYDENPMQMQMKILTEDVAFPP-------

*T. cruzi* PEFLLGQPHGRAVDWWALGILLYEMIEGIPPFFNENSNEMYEEILKGELKFGTVGDEESG

*T. brucei* PEFLLGKPHGKAVDWWSLGILLYEMLEGIPPFYSENVSAMYDKILSSELQFGDGEGGSNN

*T. vivax* PEIVLGKPHGKAVDWWSLGILLYEMLEGVPPYYNENVNAMYDKILSEELKFGTGDDE-SD

**:: * ***:*****:**::: **: . *::.** * :**. :: *

XI

_ __________________________________

*L. braziliensis* HIQITEETKDLIRRLLNKNPERRLQTLEEFKAHKCFSNLDFGLLEGCKLKAPITPDPNPA

*L. panamensis* HIQITEETKDLIRCLLNKNPERRLQTLEEFKAHKCFSNLDFGLLEGRKLKAPITPDPNPA

*L. mexicana* HIQITEETKDLIRCLLNKNPERRLQTLEAFKAHKCFSNLDFGLLEARKLKAPITPDPNPA

*L. major* HIQITEETKDLIRCLLNKNPERRLQTLEAFKAHKCFSNLDFCLLEARKLKAPITPDPNPA

*L. donovani*(I) HIQITEETKDLIRCLLNKNPERRLQTLEAFKAHKCFSNLDFGLLEARKLKAPITPDPNPA

*L. donovani*(N) HIQITEETKDLIRCLLNKNPERRLQTLEAFKAHKCFSNLDFGLLEARKLKAPITPDPNPA

*L. infantum* HIQITEETKDLIRCLLNKNPERRLQTLEAFKAHKCFSNLDFGLLEARKLKAPITPDPNPA

*T. cruzi* LPVISENAKALLRRLLDRNPQTRLQDLEEFKKHPFFEDIDWEKLSRREIQPPFRPSSNIM

*T. brucei* MPQISEEAQDLLRRLLDRNPDTRLQDVEELKGHPFFRDLDWEKLFRREIEPPFRPDGNAL

*T. vivax* IPAISEAAQDILRRLLDRDPDTRLQDLEDVKAHPFFSDIDWDKLGKREIEPPFRPNQDPF

*:* :: ::* **:::*: *** :* .* * * ::*: * ::: *: *. :
