# Supplementary material for: Identification of a RAC/AKT-like gene in Leishmania parasites as a putative therapeutic target in leishmaniasis
Source: Parasit Vectors. 2017 Oct 10;10:458. doi: 10.1186/s13071-017-2379-y (PMC5633885; doi:10.1186/s13071-017-2379-y)
Supplement: Supplementary file 3 — Analysis of RAC/AKT proteins from different origins. Multiple sequence alignment was performed using the Clustal O (1.2.4) program. Asterisks indicate identity and small dots represent similarity (DOC 50 kb) [file 13071_2017_2379_MOESM3_ESM.doc]

AKT1_HUMAN ------------------------------------------------------------

AKT2_HUMAN ------------------------------------------------------------

AKT3_HUMAN ------------------------------------------------------------

*M.musculus* ------------------------------------------------------------

*C.familiaris* ------------------------------------------------------------

*D.melanogaster* ----MNYL--PFVLQRRSTVVASAPAPGSASRIPES-------PTTTGSNIINIIYSQST 47

*S.mansoni* MEILCNYSQNPVVGSSKSSHTEHEPVSVSA-KVQDTTASQRRNINCTSSNIFSSLQ--NA 57

*C.elegans* ------------------------------------------------------------

*D.discoideum* ------------------------------------------------------------

*L.mexicana* ------------------------------------------------------------

*L.braziliensis* ------------------------------------------------------------

*L.infantum* ------------------------------------------------------------

*L.major* ------------------------------------------------------------

*L.donovani* ------------------------------------------------------------

*L.panamensis* ------------------------------------------------------------

*T.cruzi* ------------------------------------------------------------

*T.brucei* ------------------------------------------------------------

*T.vivax* ------------------------------------------------------------

AKT1_HUMAN ------------------------------------------------------MSDVAI 6

AKT2_HUMAN ------------------------------------------------------MNEVSV 6

AKT3_HUMAN ------------------------------------------------------MSDVTI 6

*M.musculus* -----------------------------AREETLIIIPG----LPLSLGATDTMNDVAI 27

*C.familiaris* ------------------------------------------------------MNDVAI 6

*D.melanogaster* HPNSSPTSGSAEKFSWQQSWPSRTSAAPTHDSGTMSINTTFDLSSPSVTSGHALTEQTQV 107

*S.mansoni* HLPVVPNPNVLFKNELTENYS---------DNLTPRISP-YLL-GMLQCRPVSLPLTRNV 106

*C.elegans* --------------------------------------------MSMTSLSTKSRRQEDV 16

*D.discoideum* ------------------------------------------------------MSTAPI 6

*L.mexicana* ------------------------------------------------------------

*L.braziliensis* ------------------------------------------------------------

*L.infantum* ------------------------------------------------------------

*L.major* ------------------------------------------------------------

*L.donovani* ------------------------------------------------------------

*L.panamensis* ------------------------------------------------------------

*T.cruzi* ---------------------------------------------------------MSV 3

*T.brucei* ---------------------------------------------------------MTI 3

*T.vivax* ---------------------------------------------------------MAA 3

AKT1_HUMAN VKEGWLHKRGE-YIKTWRPRYFLLKNDGTFIGYKERPQDVDQRE--APLNNFSVAQCQLM 63

AKT2_HUMAN IKEGWLHKRGE-YIKTWRPRYFLLKSDGSFIGYKERPEAPDQTL--PPLNNFSVAECQLM 63

AKT3_HUMAN VKEGWVQKRGE-YIKNWRPRYFLLKTDGSFIGYKEKPQDVDL-P--YPLNNFSVAKCQLM 62

*M.musculus* VKEGWLHKRGE-YIKTWRPRYFLLKNDGTFIGYKERPQDVDQRE--SPLNNFSVAQCQLM 84

*C.familiaris* VKEGWLHKRGE-YIKTWRPRYFLLKNDGTFIGYKERPQDVEQRE--SPLNNFSVAQCQLM 63

*D.melanogaster* VKEGWLMKRGE-HIKNWRQRYFVLHSDGRLMGYRSKPADSASTPSDFLLNNFTVRGCQIM 166

*S.mansoni* VKEGWLMKRGE-HIKNWRRRYFKLREDGTFYGYKIQPKDDMA----QPLNNFTVRDCQII 161

*C.elegans* VIEGWLHKKGE-HIRNWRPRYFMIFNDGALLGFRAKPKEGQPFP--EPLNDFMIKDAATM 73

*D.discoideum* KHEGFLTKEGG-GFKSWKKRWFILKGG-DLSYYKTKGELV-------PLGVIHLNTSGHI 57

*L.mexicana* -MSGYLKVLSPD--GRWETCYIEIDDA-KLRIWRTKGDKESSA---AVVKELDLK-CATL 52

*L.braziliensis* -MSGYLKVLSQD--GSWETRYIEIDNT-KLYIWRTKGDKESGA---AVVKELDLK-CATL 52

*L.infantum* -MSGYLKVLSPD--GRWETRYVEIDDA-KLRIWRTKGDKESSA---AVVKELDLK-CATL 52

*L.major* -MSGYLKVLSPD--GRWETRYIEIDDA-KLRIWRTKGDKESSA---AVVKELDLK-CATL 52

*L.donovani* -MSGYLKVLSPD--GRWETRYIEIDDA-KLRIWRTKGDKESSA---AVVKELDLK-CATL 52

*L.panamensis* -MSGYLKVLSQD--GSWETRYIEIDNT-KLYIWRTKGDKESGA---AVVKELDLK-CATL 52

*T.cruzi* EYSGYLQKTGGKFYKKNQTRYFELRGP-MLCYWKRRPSGPHVS----PTGTIDLTNARFV 58

*T.brucei* DYSGFLDEPSGASSEVTASRYFEIRGS-ILYCWTYKPENPGDK----PLSSIDLTDVHIT 58

*T.vivax* DYSGYLQKESGKFHKKKETRYFELRGT-MLYYWASRTSGLKEN----PIGSIDVTDTRIT 58

.*:: . :. : : : : : :

AKT1_HUMAN K--TERPRPNTFIIRCLQWTTVIERTFHVETPEEREEWTTAIQTVADGLKKQ--EE--EE 117

AKT2_HUMAN K--TERPRPNTFVIRCLQWTTVIERTFHVDSPDEREEWMRAIQMVANSLKQRAPGE--DP 119

AKT3_HUMAN K--TERPKPNTFIIRCLQWTTVIERTFHVDTPEEREEWTEAIQAVADRLQRQ--EE--ER 116

*M.musculus* K--TERPRPNTFIIRCLQWTTVIERTFHVETPEEREEWATAIQTVADGLKRQ--EE--ET 138

*C.familiaris* K--TERPRPNTFIIRCLQWTTVIERTFHVETPEEREEWTTAIQTVADGLKRQ--EE--EM 117

*D.melanogaster* T--VDRPKPFTFIIRGLQWTTVIERTFAVESELERQQWTEAIRNVSSRLIDVGE----VA 220

*S.mansoni* C--LNKPKPYTFLIRGLQWTNVVERLFFVETEAERNYWLSAIQSVANRLKSSFEQP--VS 217

*C.elegans* L--FEKPRPNMFMVRCLQWTTVIERTFYAESAEVRQRWIHAIESISKKYKGTNANPQEEL 131

*D.discoideum* KNSDRKKRVNGFEVQTPS----RTYFLCSETEEERAKWIEILINERELLLNGGK------ 107

*L.mexicana* RE---VSEPNTWAVQPEKA--ESTY-FQADGEERKTEWMDTLRHYNS------------- 93

*L.braziliensis* RE---VSEPNTWAVQPEKA--EATY-FQADGEGRKTEWMDTLRHYNS------------- 93

*L.infantum* RE---VSEPNTWAVQPEKA--EATY-FQADGEERKTEWMDTLRHYNS------------- 93

*L.major* RE---VSEPNTWAVQPEKA--EATY-FQADGEERKTEWMDTLRHYNS------------- 93

*L.donovani* RE---VSEPNTWAVQPEKA--EATY-FQADGEERKTEWMDTLRHYNS------------- 93

*L.panamensis* RE---VSEPNTWAVQPEKA--EATY-FQADGEGRKTEWMDTLRHYNS------------- 93

*T.cruzi* EN---PKDPRSWTIEGDHL--SKTFTLTADTEEQREAWVREMSKVKPENRE--------Q 105

*T.brucei* RD---EADRRSWSVQGGKL--RKPHMFTAENEEEREVWIEKMAHPNPANTE--------I 105

*T.vivax* RD---SKDACLWTIEGERL--RKSYTFCAENEEQRDIWVNKMKNASPTGAV--------P 105

: :. : : : * :

AKT1_HUMAN MDFRSGSP----S--DNSGAEEM-----------------------EVSLAKPKHRVTMN 148

AKT2_HUMAN MDYKCGSP----S--DSSTTEEM-----------------------EVAVSKARAKVTMN 150

AKT3_HUMAN MNCSPTSQ----I--DNIGEEEM-----------------------DASTTH-HKRKTMN 146

*M.musculus* MDFRSGSP----S--DNSGAEEM-----------------------EVSLAKPKHRVTMN 169

*C.familiaris* MDFRSGSP----S--DNSGAEEM-----------------------EVSLAKPKHRVTMN 148

*D.melanogaster* MTPSEQTDMT-DVDMATIAEDELSEQF---------------SVQGTTCNSSGVKKVTLE 264

*S.mansoni* VHNL------------NLAENMI-----------------------VDIPQRPVKRYSVN 242

*C.elegans* METNQQPKIDEDSEFAGAAHAIMGQPSSGHGDNCSIDFRASMISIADTSEAAKRDKITME 191

*D.discoideum* -------------------------------------------------QPKKSEKVGVA 118

*L.mexicana* -------------------------------------------------GSTGSEKVTLR 104

*L.braziliensis* -------------------------------------------------SSIGSEKVTLS 104

*L.infantum* -------------------------------------------------SSSASEKVTLR 104

*L.major* -------------------------------------------------SSSGSEKVTLR 104

*L.donovani* -------------------------------------------------SSSASEKVTLR 104

*L.panamensis* -------------------------------------------------SNIGSEKVTLR 104

*T.cruzi* PSTAVQSS----------GD-----------------------VHAVTLYTGGRHKVSLD 132

*T.brucei* LRTLEQTS----------DG-----------------------DDTAPLCSGGSNRVSLN 132

*T.vivax* LDVIDVSS----------EE-----------------------EEAATL--YGDDKVSLN 130

: :

AKT1_HUMAN EFEYLKLLGKGTFGKVILVKEKATGRYYAMKILKKEVIVAKDEVAHTLTENRVLQNSRHP 208

AKT2_HUMAN DFDYLKLLGKGTFGKVILVREKATGRYYAMKILRKEVIIAKDEVAHTVTESRVLQNTRHP 210

AKT3_HUMAN DFDYLKLLGKGTFGKVILVREKASGKYYAMKILKKEVIIAKDEVAHTLTESRVLKNTRHP 206

*M.musculus* EFEYLKLLGKGTFGKVILVKEKATGRYYAMKILKKEVIVAKDEVAHTLTENRVLQNSRHP 229

*C.familiaris* EFEYLKLLGKGTFGKVILVKEKATGRYYAMKILKKEVIVAKDEVAHTLTENRVLQNSRHP 208

*D.melanogaster* NFEFLKVLGKGTFGKVILCREKATAKLYAIKILKKEVIIQKDEVAHTLTESRVLKSTNHP 324

*S.mansoni* DFRLLKVLGKGTFGKVILCQENETGHFYAMKILKKSVLIEKEEVVHTMTENRVLQQCKHP 302

*C.elegans* DFDFLKVLGKGTFGKVILCKEKRTQKLYAIKILKKDVIIAREEVAHTLTENRVLQRCKHP 251

*D.discoideum* DFELLNLVGKGSFGKVIQVRKKDTGEVYAMKVLSKKHIVEHNEVEHTLSERNILQKINHP 178

*L.mexicana* DFEKKFVLGKGSYGKVFMVVKKDTDKWYAMKEMSAEKMRQAE-IKAPFAERIILEEIDHP 163

*L.braziliensis* DFEKKFVLGKGSYGKVFMVVKKDTDKWYAMKEMSAEKMRQAE-IKAPFAERIILEEIDHP 163

*L.infantum* DFEKKFVLGKGSYGKVFMVVKKDTDKWYAMKEMSAEKMRQAE-IKAPFAERIILEEIDHP 163

*L.major* DFEKKFVLGKGSYGKVFMVVKKDTDKWYAMKEMSAEKMRQAE-IKAPFAERIILEEIDHP 163

*L.donovani* DFEKKFVLGKGSYGKVFMVVKKDTDKWYAMKEMSAEKMRQAE-IKAPFAERIILEEIDHP 163

*L.panamensis* DFEKKFVLGKGSYGKVFMVVKKDTDKWYAMKEMSAEKMRQAE-IKAPFAERIILEEIDHP 163

*T.cruzi* DFELKATIGAGSFSNVFVAREKSTDKVYAIKEMGKELIQQHNMLSNIAAEKHILQTISHP 192

*T.brucei* DFQFAAKIGKGSFSSVYAATEKATGKTYAIKKMEKEVIERYNMIDNISAERLILQKIDHP 192

*T.vivax* DFEVTTTIGKGSFSFVYAAREKSTNKLYAVKEMKKEVIERANMLENIFTEKRILQDIRHP 190

:* :* *::. * :: : . **:* : . : : : :* :*: **

AKT1_HUMAN FLTALKYSFQTHDRLCFVMEYANGGELFFHLSRERVFSEDRARFYGAEIVSALDYLHSEK 268

AKT2_HUMAN FLTALKYAFQTHDRLCFVMEYANGGELFFHLSRERVFTEERARFYGAEIVSALEYLHSRD 270

AKT3_HUMAN FLTSLKYSFQTKDRLCFVMEYVNGGELFFHLSRERVFSEDRTRFYGAEIVSALDYLHSGK 266

*M.musculus* FLTALKYSFQTHDRLCFVMEYANGGELFFHLSRERVFSEDRARFYGAEIVSALDYLHSEK 289

*C.familiaris* FLTALKYSFQTHDRLCFVMEYANGGELFFHLSRERVFPEDRARFYGAEIVSALDYLHSEK 268

*D.melanogaster* FLISLKYSFQTNDRLCFVMQYVNGGELFWHLSHERIFTEDRTRFYGAEIISALGYLHSQG 384

*S.mansoni* FMTELRYSFTTPNYLCFVMEYVNGGELFFHLQRDRVFSEERAKFYGAEITLALGYLHHQN 362

*C.elegans* FLTELKYSFQEQHYLCFVMQFANGGELFTHVRKCGTFSEPRARFYGAEIVLALGYLHRCD 311

*D.discoideum* FLVNLNYSFQTEDKLYFILDYVNGGELFYHLQKDKKFTEDRVRYYGAEIVLALEHLHLSG 238

*L.mexicana* FIVHLHYSFQEQGNLYMILDLLAGGELFTYIEQHAPLDEEVVKFYAAEVALALGYLHSRN 223

*L.braziliensis* FIVHLHYSFQEQGNLYMILDLLAGGELFTYIEQHAPLDEEVVKFYAAEVALALGYLHSRN 223

*L.infantum* FIVHLHYSFQEQGNLYMILDLLAGGELFTYIEQHAPLDEEVVKFYAAEVALALGYLHSRN 223

*L.major* FIVHLHYSFQEQGNLYMILDLLAGGELFTYIEQHAPLDEEVVKFYAAEVALALGYLHSRN 223

*L.donovani* FIVHLHYSFQEQGNLYMILDLLAGGELFTYIEQHAPLDEEVVKFYAAEVALALGYLHSRN 223

*L.panamensis* FIVHLHYSFQEQGNLYMILDLLAGGELFTYIEQHAPLDEEVVKFYAAEVALALGYLHSRN 223

*T.cruzi* FIVSLHYAFETKKCLYLVLDFLPGGELFFHLAKEKVFDEYRAKFYCGEIALAIGYLHSLD 252

*T.brucei* FIVSLHYAFQTKGSLYLVMDFLSGGELFFHLESVSVFDEWRAKFYCGEIALALGYLHAQD 252

*T.vivax* FIVSLHYAFQTPNCLYLVMDFLPGGELFFHLGNVKTFDEQRAKFYCGEIALALEYLHEHN 250

*: *.*:* * :::: ***** :: : * .::* .*: *: :**

AKT1_HUMAN NVVYRDLKLENLMLDKDGHIKITDFGLCKEGIKD-GATMKTFCGTPEYLAPEVLEDNDYG 327

AKT2_HUMAN -VVYRDIKLENLMLDKDGHIKITDFGLCKEGISD-GATMKTFCGTPEYLAPEVLEDNDYG 328

AKT3_HUMAN -IVYRDLKLENLMLDKDGHIKITDFGLCKEGITD-AATMKTFCGTPEYLAPEVLEDNDYG 324

*M.musculus* NVVYRDLKLENLMLDKDGHIKITDFGLCKEGIKD-GATMKTFCGTPEYLAPEVLEDNDYG 348

*C.familiaris* NVVYRDLKLENLMLDKDGHIKITDFGLCKEGIKD-GATMKTFCGTPEYLAPEVLEDNDYG 327

*D.melanogaster* -IIYRDLKLENLLLDKDGHIKVADFGLCKEDITY-GRTTKTFCGTPEYLAPEVLDDNDYG 442

*S.mansoni* -VVYRDLKLENLLLDKDGHIKIADFGLCKEDMYY-GASTKTFCGTPEYLAPEVLLDNDYG 420

*C.elegans* -IVYRDMKLENLLLDKDGHIKIADFGLCKEEISF-GDKTSTFCGTPEYLAPEVLDDHDYG 369

*D.discoideum* -VIYRDLKPENLLLTNEGHICMTDFGLCKEGLLTPTDKTGTFCGTPEYLAPEVLQGNGYG 297

*L.mexicana* -IIYRDLKPENVVFDRDGHACLTDFGLAKANVHE--PNAVTYCGTNEYLAPELLKGVPHG 280

*L.braziliensis* -IIYRDLKPENVVFDHEGHACLTDFGLAKANVHE--PNAVTYCGTNEYLAPELLKGVPHG 280

*L.infantum* -IIYRDLKPENVVFDRDGHACLTDFGLAKANVHE--PNAVTYCGTNEYLAPELLKGVPHG 280

*L.major* -IIYRDLKPENVVFDRDGHACLTDFGLAKANVHE--PNAVTYCGTNEYLAPELLKGVPHG 280

*L.donovani* -IIYRDLKPENVVFDRDGHACLTDFGLAKANVHE--PNAVTYCGTNEYLAPELLKGVPHG 280

*L.panamensis* -IIYRDLKPENVVFDHEGHACLTDFGLAKANVHE--PNAVTYCGTNEYLAPELLKGVPHG 280

*T.cruzi* -IIFRDLKPENIVLDEDGHACLTDFGLAKMNVSN--ASNFTFCGTTEYIAPEFLLGQPHG 309

*T.brucei* -IIYRDLKPENAVLDADGHVCLTDFGLAKMDVRD--ACNFTFCGTPEYIAPEFLLGKPHG 309

*T.vivax* -IIYRDLKPENAVLDADGHVCLTDFGLAKMVVSD--ASNFTFCGTPEYVAPEIVLGKPHG 307

:::**:* ** :: :** ::****.* : *:*** **:***.: . :*

AKT1_HUMAN RAVDWWGLGVVMYEMMCGRLPFYNQDHEKLFELILMEEIRFPR---------TLGPEAKS 378

AKT2_HUMAN RAVDWWGLGVVMYEMMCGRLPFYNQDHERLFELILMEEIRFPR---------TLSPEAKS 379

AKT3_HUMAN RAVDWWGLGVVMYEMMCGRLPFYNQDHEKLFELILMEDIKFPR---------TLSSDAKS 375

*M.musculus* RAVDWWGLGVVMYEMMCGRLPFYNQDHEKLFELILMEEIRFPR---------TLGPEAKS 399

*C.familiaris* RAVDWWGLGVVMYEMLCGRLPFYNQDHEKLFELILMEELRFPR---------TLSPEAKS 378

*D.melanogaster* QAVDWWGTGVVMYEMICGRLPFYNRDHDVLFTLILVEEVKFPR---------NITDEAKN 493

*S.mansoni* RSVDWWGLGVVMYEMMCGRLPFYSSDHEVLFELILQENVSFPA---------RLSHPAQD 471

*C.elegans* RCVDWWGVGVVMYEMMCGRLPFYSKDHNKLFELIMAGDLRFPS---------KLSQEART 420

*D.discoideum* KQVDWWSFGSLLYEMLTGLPPFYNQDVQEMYRKIMMEKLSFPH---------FISPDARS 348

*L.mexicana* KAVDWWSLGLMMCEMLFNDLPFYDENPMQMQMKILTEDVAFPP-------HIQITEETKD 333

*L.braziliensis* KAVDWWSLGLMMCEMLFNDLPFYDENPMQMQMKILTEDVSFPS-------HIQITEETKD 333

*L.infantum* KAVDWWSLGLMMCEMLFNDLPFYDENPMQMQMKILTEDVAFPP-------HIQITEETKD 333

*L.major* KAVDWWSLGLMMCEMLFNDLPFYDENPMQMQMKILTEDVAFPP-------HIQITEETKD 333

*L.donovani* KAVDWWSLGLMMCEMLFNDLPFYDENPMQMQMKILTEDVAFPP-------HIQITEETKD 333

*L.panamensis* KAVDWWSLGLMMCEMLFNDLPFYDENPMQMQMKILTEDVAFPS-------HIQITEETKD 333

*T.cruzi* RAVDWWALGILLYEMIEGIPPFFNENSNEMYEEILKGELKFGDVGGEESGLPVISENAKA 369

*T.brucei* KAVDWWSLGILLYEMLEGIPPFYSENVSAMYDKILSSELQFGDGEGGSNNMPQISEEAQD 369

*T.vivax* KAVDWWSLGILLYEMLEGVPPYYNENVNAMYDKILSEELKFGTGDDE-SDIPAISEAAQD 366

: ****. * :: **: . *::. : : *: .: * : ::

AKT1_HUMAN LLSGLLKKDPKQRLGGGSEDAKEIMQHRFFAGIVWQHVYEKKLSPPFKPQVTSETDTRYF 438

AKT2_HUMAN LLAGLLKKDPKQRLGGGPSDAKEVMEHRFFLSINWQDVVQKKLLPPFKPQVTSEVDTRYF 439

AKT3_HUMAN LLSGLLIKDPNKRLGGGPDDAKEIMRHSFFSGVNWQDVYDKKLVPPFKPQVTSETDTRYF 435

*M.musculus* LLSGLLKKDPTQRLGGGSEDAKEIMQHRFFANIVWQDVYEKKLSPPFKPQVTSETDTRYF 459

*C.familiaris* LLSGLLKKDPKQRLGGGSEDAKEIMQHRFFASIVWQDVYEKKLSPPFKPQVTSETDTRYF 438

*D.melanogaster* LLAGLLAKDPKKRLGGGKDDVKEIQAHPFFASINWTDLVLKKIPPPFKPQVTSDTDTRYF 553

*S.mansoni* ILSRLLIKDPTSRLGGGIQDVLEVMAHLFFASVDWDRLIRKDIQPPWKPDVVDEKDTKYV 531

*C.elegans* LLTGLLVKDPTQRLGGGPEDALEICRADFFRTVDWEATYRKEIEPPYKPNVQSETDTSYF 480

*D.discoideum* LLEQLLERDPEKRLA----DPNLIKRHPFFRSIDWEQLFQKNIPPPFIPNVKGSADTSQI 404

*L.mexicana* LIRCLLNKNPERRLQ----TLEAFKAHKCFSNLDFGLLEARKLKAPITPDPN---PAHNF 386

*L.braziliensis* LIRRLLNKNPERRLQ----TLEEFKAHKCFSNLDFGLLEGCKLKAPITPDPN---PAHNF 386

*L.infantum* LIRCLLNKNPERRLQ----TLEAFKAHKCFSNLDFGLLEARKLKAPITPDPN---PAHNF 386

*L.major* LIRCLLNKNPERRLQ----TLEAFKAHKCFSNLDFCLLEARKLKAPITPDPN---PAHNF 386

*L.donovani* LIRCLLNKNPERRLQ----TLEAFKAHKCFSNLDFGLLEARKLKAPITPDPN---PAHNF 386

*L.panamensis* LIRCLLNKNPERRLQ----TLEEFKAHKCFSNLDFGLLEGRKLKAPITPDPN---PAHNF 386

*T.cruzi* LLRRLLDRNPQTRLQ----DLEEFKKHPFFEDIDWVKLSRREIQPPFRPSSN---ILCNF 422

*T.brucei* LLRRLLDRNPDTRLQ----DVEELKGHPFFRDLDWEKLFRREIEPPFRPDGN---ALSNF 422

*T.vivax* ILRRLLDRDPDTRLQ----DLEDVKAHPFFSDIDWDKLGKREIEPPFRPNQD---PFSNF 419

:: ** ::* ** . * : : .: * *. .

AKT1_HUMAN DEEFTAQMITITPPDQ--DDSMECVDSERRPHFPQFSYSASGTA---------------- 480

AKT2_HUMAN DDEFTAQSITITPPDRYDSLGL--LELDQRTHFPQFSYSASIRE---------------- 481

AKT3_HUMAN DEEFTAQTITITPPEKYDEDGMDCMDNERRPHFPQFSYSASGRE---------------- 479

*M.musculus* DEEFTAQMITITPPDQ--DDSMECVDSERRPHFPQFSYSASGTA---------------- 501

*C.familiaris* DEEFTAQMITITPPDQ--GDNMEGEDSERRPHFPQFSYSASGTA---------------- 480

*D.melanogaster* DKEFTGESVELTPPDPTGPLGS----IAEEPLFPQFSYQGDMASTLGTSSHISTSTSLAS 609

*S.mansoni* PDEFKDTSVDLTPPNDNEDNM---NRIVDGPYFEQFSFHGSRQSLNSRVSGYSFGDTF-- 586

*C.elegans* DNEFTSQPVQLTPPSRSGALATVDEQEEMQSNFTQFSFHNVMGSINRIHEASEDNEDYD- 539

*D.discoideum* DPVFTDEAPSLTMAGEC------ALNPQQQKDFEGFTYVAESEHLR-------------- 444

*L.mexicana* AKEFTSEVIV--QNESP---------SEAVVTLAGYTYDRDLSEQEKSPSHSPTIAEELR 435

*L.braziliensis* AKEFTSEVIV--QNESP---------SQAIVTLAGYTYDRDSSEQEKSPSHSPTIAEELR 435

*L.infantum* AKEFTSEVIV--QNESP---------SQAVVTLAGYTYDRDLSEQEKSPSHSPTIAEELR 435

*L.major* AKEFTSEVIV--QNESP---------SQAVVTLAGYTYDRDLSEQEKSPSHSPTIAEELR 435

*L.donovani* AKEFTSEVIV--QNESP---------SQAVVTLAGYTYDRDLSEQEKSPSHSPTIAEELR 435

*L.panamensis* AKEFTSEVIV--QNESP---------SQAIVTLAGYTYDRDSSEQEKSPSHSPTIAEELR 435

*T.cruzi* DEDFTSKEPRAGFQEED---------GGEHGNIACFSFDGQMGPA--------------- 458

*T.brucei* DQEFTSADPPMVQPDDE---------VVEDKSICGFTFNGRSRPT--------------- 458

*T.vivax* DSEFTSLAPQIARHNEQ---------CREGADVAGFTFVGNRGTM--------------- 455

*. . :::

AKT1_HUMAN ------------------------------------------------------------

AKT2_HUMAN ------------------------------------------------------------

AKT3_HUMAN ------------------------------------------------------------

*M.musculus* ------------------------------------------------------------

*C.familiaris* ------------------------------------------------------------

*D.melanogaster* MQ---------------------------------------------------------- 611

*S.mansoni* ------------------------------------------------------------

*C.elegans* MG---------------------------------------------------------- 541

*D.discoideum* ------------------------------------------------------------

*L.mexicana* QRRASKKSFTNGSDAASSPVTGENGTLSSSPAGAPTKQAAAVPVKKVEHHIPSKVTPQAV 495

*L.braziliensis* QRRASKKTSSSGSEAVSPPVTGGKRTSNSSSAGASAKQAATGPIKKVEHHIPAKVAPQAA 495

*L.infantum* QRRASMKKSTNGSDAASPPVTGENRTSNSSPAGAPTKQAAAGPVKKVEHHIPAKVAPQAA 495

*L.major* QRRASKKRPTNGSDAASPPVTGENRTSNSSPAGVPMKQAAPGPVKKVEHHIPAKVAPQAA 495

*L.donovani* QRRASMKKSTNGSDAASPPVTGENRTSNSSPAGAPTKQAAAGPVKKVEHHIPAKVAPQAA 495

*L.panamensis* QRRASKKTSSSGSEAVSPPVTGGKRTSNSSSAGASAKQAATGPIKKVEHHIPAKVAPQAA 495

*T.cruzi* ------------------------------------------------------------

*T.brucei* ------------------------------------------------------------

*T.vivax* ------------------------------------------------------------

AKT1_HUMAN ---------------

AKT2_HUMAN ---------------

AKT3_HUMAN ---------------

*M.musculus* ---------------

*C.familiaris* ---------------

*D.melanogaster* ---------------

*S.mansoni* ---------------

*C.elegans* ---------------

*D.discoideum* ---------------

*L.mexicana* RKKLTGNKSFDKPTK 510

*L.braziliensis* RKKLTQNSSFDKAAK 510

*L.infantum* RKKLTGNKSFDKPTK 510

*L.major* RKKLTGNKSFDKPTK 510

*L.donovani* RKKLTGNKSFDKPTK 510

*L.panamensis* RKKLTQNSSFDKPTK 510

*T.cruzi* ---------------

*T.brucei* ---------------

*T.vivax* ---------------
